# Japanese Local Governments’ Dissemination Activities for Advance Care Planning: A Descriptive Analysis of a Nationwide Survey during the COVID-19 Pandemic

**DOI:** 10.3390/ijerph20075408

**Published:** 2023-04-05

**Authors:** Noriko Morioka, Masayo Kashiwagi, Ako Machida, Kyoko Hanari, Takehiro Sugiyama, Ryota Inokuchi, Nanako Tamiya

**Affiliations:** 1 Department of Nursing Health Service Research, Graduate School of Health Care Sciences, Tokyo Medical and Dental University, Tokyo 1138510, Japan; 2 Health Services Research and Development Center, University of Tsukuba, Tsukuba 3058575, Japan; 3 Institute for Global Health Policy Research, Bureau of International Health Cooperation, National Center for Global Health and Medicine, Tokyo 1628655, Japan; 4 Diabetes and Metabolism Information Center, Research Institute, National Center for Global Health and Medicine, Tokyo 1628655, Japan; 5 Department of Health Services Research, Institute of Medicine, University of Tsukuba, Tsukuba 3058575, Japan

**Keywords:** advance care planning, end of life care, local government policy

## Abstract

This study aims to compare the awareness-raising activities between municipalities with and without focused anti-infection measures during the 2019 coronavirus disease (COVID-19) pandemic. Descriptive analysis was conducted using a nationwide self-administered questionnaire survey on municipalities’ activities for residents and for healthcare providers and care workers (HCPs) in October 2022 in Japan. This study included 433 municipalities that had conducted awareness-raising activities before 2019 Fiscal Year. Workshops for residents were conducted in 85.2% of the municipalities, and they were more likely to be conducted in areas with focused anti-infection measures than those without measures (86.8% vs. 75.4%). Additionally, 85.9% of the municipalities were impacted by the pandemic; 50.1% canceled workshops, while 26.0% switched to a web-based style. Activities for HCPs were conducted in 55.2–63.7% of the municipalities, and they were more likely to be conducted in areas with focused anti-infection measures. A total of 50.6–62.1% of the municipalities changed their workshops for HCPs to a web-based style. Comparisons between areas with and without focused anti-infection measures indicated that the percentages of those impacted for all activities were not significantly different. In conclusion, awareness-raising activities in municipalities were conducted with new methods during the COVID-19 pandemic. Using information technology is essential to further promote such activities for residents.

## 1. Introduction

Advanced care planning (ACP) is “a process that supports adults at any age or stage of health in understanding and sharing their values, life goals, and preferences regarding future medical care [1]”. For the past two decades, ACP has been of great interest, and numerous studies have investigated its impact in various settings and populations [2,3], such as those with older adults [4], as well as dementia [5], and heart failure [6]. The COVID-19 pandemic, which started in January 2020, with the first wave peaking in Japan on 11 April 2020, highlighted the importance of ACP for older adults, who are at an increased risk of mortality due to COVID-19, even with prolonged life support and they experience a substantially lower quality of life after those treatments conclude [7,8]. During the pandemic, it is recommended that healthcare providers discuss the goals of care with older patients that have chronic diseases in order to avoid unhelpful and unwanted high-intensity treatments under the limited healthcare resources available due to the pandemic [7].

In Japan, a case in 2006, whereby a physician removed a ventilator from an unconscious patient without clarification of the patient’s intention raised discussion about decision-making in end-of-life (EOL) care [9]. The Ministry of Health, Labour, and Welfare (MHLW) introduced guidelines on the decision-making process for EOL care in 2007, including the rules for starting and stopping life-prolonging measures, such as ventilators and cardiac massage in hospital settings. According to the spread of the concept of ACP in European countries [10] and the community-based integrated care system that enables people to live in familiar places at the EOL stage, the MHLW hosted an expert committee discussing the decision-making process on EOL care and revised guidelines in 2018 [11]. The main changes in the revised guidelines were that they specify a decision-making process, respect for the person’s wishes, and the involvement of a multidisciplinary team. In expert committee reports, municipalities are supposed to raise awareness for community residents and health and care providers, and care workers (HCPs) about ACP based on the guidelines [9]. Municipalities are responsible for hosting workshops, individual consultation services at municipal offices, and providing information to community residents regarding EOL healthcare and long-term care via public relations magazines. Additionally, municipalities are responsible for hosting workshops, and discussion and review meetings among HCPs and local government officers. As of 2017, approximately 40% of the municipalities in Japan conducted awareness-raising activities regarding EOL care [12]. Since it is indicated that ACP in Japan needs to be more sophisticated through future policy and educational program enhancements [13], awareness-raising public policy needs to be further promoted in the municipalities closest to the inhabitants.

However, the impact of COVID-19 on ACP awareness-raising activities in different municipalities is not clear. It is possible that the increasing prevalence of sudden deaths due to COVID-19 have made community residents, especially older adults, and their family members, more aware of the importance of ACP. Consequently, the need for municipality dissemination activities for ACP might be increasing. On the other hand, with the COVID-19 pandemic, municipalities have been overloaded with work, as they are responsible for applying for subsidies, such as unemployment compensation owing to the pandemic, and for coordinating COVID-19 vaccinations. Simultaneously, measures to prevent infection, such as reducing face-to-face contact hours, have been implemented. The COVID-19 pandemic has disrupted and paralyzed municipal operations. Therefore, it is not surprising that the awareness-raising activities by municipalities, which are only starting, have also been significantly affected by COVID-19.

In this context, this study aimed to investigate whether the COVID-19 pandemic would be a barrier or a promotion factor for the ACP awareness-raising activities by the municipalities.

## 2. Materials and Methods

### 2.1. Study Design and Materials

We performed a secondary analysis using nationwide, self-administered questionnaire survey data from municipalities in Japan and other open-source administrative data. The survey was conducted in October 2022 by the MHLW and the project team to investigate the current situation of municipal activities related to ACP awareness-raising in the 2022 fiscal year (FY). Among all 1741 municipalities, local government officers in charge of such activities were invited to complete the web-based questionnaire. It contained the following items: budget for the policy, contents of activities, the impact of the COVID-19 pandemic on the activities, adherence to the national guidelines, and issues for promoting community awareness-raising for EOL decisions. Questionnaire items were developed based on a previous survey in 2007 and the municipality’s role in the MHLW expert committee report [9]. To raise awareness of ACP, the report stated that the municipality needs to conduct workshops for residents and HCPs and provide information to community residents concerning healthcare and long-term care at EOL. Content validity was discussed by a research team comprising several nursing researchers, physicians, and health service researchers. The survey was anonymous, and participants signed an informed consent form on the first page of the online survey to participate in the study.

Of the 1741 municipalities, 912 were returned (valid response rate: 52.4%). Among the respondents, 433 municipalities (47.5%) indicated that they introduced awareness-raising activities at least once before the 2019 FY, which means before the COVID-19 pandemic. In this study, these 433 municipalities were included to investigate the impact of the COVID-19 pandemic on awareness-raising activities.

### 2.2. Outcomes

Our outcomes were the following: awareness-raising activities by municipalities in 2022 FY and the impact of the COVID-19 pandemic thereon. According to MHLW expert committee reports [9], awareness-raising activities were categorized into two groups (A) activities for community residents and (B) activities for HCPs in this study. (A) There were three activities for community residents: workshops for residents, individual consultation services at municipal offices, and information to community residents concerning healthcare and long-term care at EOL via public relations magazine. (B) There were three activities for HCPs: workshops for healthcare providers; workshops for care workers; and discussion and review meetings among HCPs and local government officers. All answers to the questions were binary (yes or no). For the above activities, we asked, “do you conduct the activities in 2022 FY? (yes or no)” and “if yes, is there any kind of impact from the COVID-19 pandemic on the activity (yes or no)”, and “if yes, what was the impact (free description)”.

### 2.3. Characteristics of Municipalities

As characteristics of municipalities, we obtained the following data from the open-source national census: total population; percentage of people aged≥ 65, from the Annual Report on Internal Migration in Japan, derived from the basic resident registration, as of 2022 [14]; the financial index, of which a higher number indicates more finances, from the website of the Ministry of Internal Affairs and Communications [15]; the number of hospitals; the number of home care providers dispensing home care; the number of home-visiting nursing agencies; the number of beds in long-term-care facilities; and the percentage of home deaths in 2021, from the website of the MHLW [16].

As variables of the degree of COVID-19 outbreaks in each municipality, we obtained the focused anti-infection measures for COVID-19 cases, as of January 2022, from the website of the Cabinet Secretariat [17]. Pursuant to the Act on Special Measures for Pandemic Influenza and New Infectious Diseases Preparedness and Response (Act No. 31 of 2012), the government of Japan declared a four-tiered alert system [17]. A state of emergency can be declared at the prefecture-level when the infection situation reaches the highest level on the government’s four-tiered alert system. Afterward, the government introduced a new policy on 5 April 2021, focusing anti-infection measures on the specific areas (there is no specific definition of the area, i.e., prefecture-level or several municipalities or one municipality level or several municipalities) corresponding to stages two or three. Stage two is a situation, whereby the number of newly infected patients is rapidly increasing in the region, although the increase can be handled by increasing the number of hospital beds. Stage three comprises an inability to provide medical care for COVID-19 patients without restrictions on general medical care. In the survey period, some prefectures and/or municipalities imposed focused anti-infection measures from the beginning of January 2022 to the end of March 2022.

### 2.4. Statistical Analysis

Numeric variables are presented as medians and interquartile ranges (IQR, 25th/75th percentiles). In contrast, categorical variables, such as municipality category (city, ward, town, and village) and awareness-raising activities, are presented as numbers and percentages. To determine the degree of COVID-19 spread within a municipality, Chi-squared tests or Fisher’s exact tests were conducted between areas under focused anti-infection measures and areas without measures. We also performed a qualitative analysis of the free description of details on the impact of the COVID-19 pandemic. In the qualitative analysis, one researcher (A.M.) extracted the codes from the free description. Another researcher (N.M.) reviewed the codes and obtained a consensus by discussing any inconsistencies. These codes were categorized based on their similarities by three researchers (A.M., N.M., and M.K.).

### 2.5. Ethical Considerations

This study was conducted in accordance with the Declaration of Helsinki (http://www.med.or.jp/dl-med/wma/helsinki2013e.pdf accessed on 3 March 2023) and the protocol was approved by the Medical Research Ethics Committee of Tokyo Medical and Dental University (No. C2022-017). Participants signed an informed consent form to participate in the study.

## 3. Results

Among the 433 municipalities, the median population was 48,091, and the median percentage of persons aged over 65 was 31.4% (Table 1). As of January 2022, 372 (85.9%) municipalities had been under-focused anti-infection measures for the COVID-19 pandemic. Our study participants had a larger population than those in all 1741 municipalities; however, other characteristics were almost identical to those in all 1741 municipalities (Appendix A, Table A1).

Table 2 presents the percentage of municipalities that conducted each awareness-raising activity among the 433 municipalities: 85.2% conducted workshops for residents, 56.8% provided individual consultation support to residents for EOL healthcare and long-term care, 65.4% provided residents with information regarding home healthcare, 57.3% conducted workshops for healthcare providers, 63.7% conducted workshops for care workers, and 55.2% conducted discussion and review meetings among HCPs and local government officers, respectively. Compared to the municipalities without focused anti-infection measures, municipalities with such measures were more likely to conduct workshops for residents and awareness-raising activities for HCPs.

Table 3 shows the percentage of municipalities that felt the impact of the COVID-19 pandemic on their awareness-raising activities, alongside a comparison of the implementation of focused anti-infection measures. Regarding the overall impact of the COVID-19 pandemic on municipal activities, 85.9% of the municipalities conducted workshops for residents, 17.1% of municipalities provided individual consultation services, 14.1% of municipalities provided residents with information regarding home healthcare, 86.7% of municipalities conducted workshops for healthcare providers, 84.1% of municipalities conducted workshops for care workers, and 81.6% conducted in municipalities discussion and review meetings among HCP and local government officers (Table 3). Comparisons between municipalities with and without focused anti-infection measures indicated that the percentages of those impacted were not significantly different for all the activities.

From the qualitative analysis of the free description of the impact of the COVID-19 pandemic on workshop-style activities, the following codes were extracted: 444 codes from 369 municipalities in workshops for residents, 277 codes from 248 municipalities in workshops for healthcare providers, 307 codes from 276 municipalities in workshops for care workers, and 269 codes from 239 municipalities in discussion and review meetings among HCPs and municipal officers. From these codes, two categories, “canceled” and “conducted with some changes”, were identified. “Conducted with some changes” included two subcategories, “conducted face-to-face with some changes” and “conducted by methods other than face-to-face” (Table 4). Furthermore, 50.1% of the municipalities mentioned canceled codes in the category workshops for residents, followed by 37.2% in discussions and review meetings among HCPs and municipal officers, 27.2% in workshops for care workers, and 23.8% for workshops for healthcare providers, in descending order. The percentage of “conducted face-to-face with some changes” was 44.2% for workshops for residents, while it was less than 30% for workshops for healthcare providers, care workers, and discussion and review meetings among HCPs and municipal officers. The percentage of “conducted by methods other than face-to-face” exceeded 50% for workshops for healthcare providers, care workers, and discussion and review meetings among HCPs and municipal officers, while it was 26.0% for workshops for residents (Table 4).

## 4. Discussion

Using the nationwide questionnaire survey data, we examined whether the extra workload of municipal officers due to the COVID-19 pandemic would limit awareness-raising activities by municipalities for EOL decision-making or whether those activities would continue to meet the increasing needs of ACP among residents. The results revealed two main findings; first, although workshops for residents were implemented in many municipalities, more than half of them were canceled due to the pandemic, and the transition to web-based workshops has not progressed. Second, only half the municipalities conducted activities for HCPs during the COVID-19 pandemic. They successfully maintained the hosting of workshops through substitute methods, such as web-based workshops.

### 4.1. Activities for Residents

Regarding the impact of the COVID-19 pandemic on the awareness-raising activities by municipalities, it is unclear whether the COVID-19 pandemic would be a barrier or a promotion factor of the activities. Although municipal officials were probably too busy with their COVID-19 duties to have time to provide EOL support, more than 85% of municipalities conducted workshops for residents. In addition, a higher percentage of municipalities with focused anti-infection measures, where COVID-19 had a significant impact on society, conducted workshops than municipalities that were not taking measures. This may be due to the increased need for promotion brought on by areas with higher rates of infected people and more instances where EOL care is offered, as COVID-19 made older individuals more vulnerable to poorer outcomes and death [18,19]. These facts were reported daily on public television and in the newspapers, and it is possible that the pandemic was an opportunity for people, especially older adults, to contemplate their own or their relatives’ deaths.

However, more than half of the municipalities canceled the workshops for residents at least once, and a few were held online regardless of whether focused anti-infection measures were conducted in the area. This might be due to the combination of the national campaign to prevent the spread of COVID-19 and the vulnerability of the target population. Since 2020, the Japanese government has promoted the “avoid the 3Cs (closed spaces, crowded places, and close-contact settings) nationwide campaign” to prevent COVID-19 outbreaks [20]; thus, several face-to-face events were canceled. Municipal activities that entail the gathering and conversing of people in close contact have also been affected regardless of the severity of COVID-19 outbreaks in each municipality.

Given the COVID-19 pandemic, it has been reported that only 40% of community-dwelling individuals [21] and 60% of older adults living with diseases [22] have discussed their decision-making on EOL in Japan. With the increased interest due to the pandemic, municipalities need to ensure awareness campaigns on ACP. It is necessary to promote efforts while sharing pioneering trials among municipalities, such as creating older people friend-web-based methods for holding events. Additionally, various publicly available interactive web-based tools have recently been developed [23]. In local governments’ future policies, the utilization of information technology will greatly help promote ACP.

### 4.2. Activities for HCPs

The second finding is that only about half of the municipalities conducting activities for HCPs, especially those under focused anti-infection measures, are more likely to plan these activities. This is similar to findings on workshops for residents. It is a fact that the need for ACP has been increasing in these areas. To promote ACP, municipalities should develop a collaborative support system through which interdisciplinary HCPs can improve their understanding of each occupation through training [24]. It is necessary to take actions that encourage the implementation of activities for HCP.

Regarding the impact of the COVID-19 pandemic, the percentage of municipalities that felt an impact on their activities was similar between areas with focused anti-infection measures and those without. Due to the impact of COVID-19 on activities for HCPs, a small percentage of municipalities that had already conducted activities canceled workshops, and more than half of those transitioning from face-to-face to web-based events, or the distribution of videos or DVDs. This was in contrast to the findings for residents where more than half of the municipalities canceled workshops, and only a small percentage change to web-based events. This result may indicate that municipalities, which were able to hold events, even under the COVID-19 pandemic, had decided from the beginning to hold web-based events for HCPs from the perspective of infection prevention, regardless of the number of infected people in the area. HCPs and system-related barriers to ACP in Asian countries are the following: HCPs’ limited knowledge and skills regarding EOL care, HCPs’ uneasiness with regard to conducting ACP, HCPs’ uneasiness with regard to conducting ACP, and lack of training and education related to ACP [25]. Additionally, HCPs are encouraged to undergo training to promote patients’ autonomy in the ACP process [26], since people in Japan typically prioritize making decisions based on experts’ opinions rather than their own [13]. It is preferable that HCP should acquire knowledge of ACP and multidisciplinary team building before they are suddenly faced with EOL care decisions for patients during a pandemic. Even under the disruptions in medical and long-term care facilities caused by the COVID-19 pandemic, it is worthwhile to promote training for HCP’s by local governments. Additionally, face-to-face relationships among multidisciplinary professionals are vital in promoting ACP and palliative care [27], and web-based workshops need to be designed to encourage the same effectiveness of relationships as face-to-face workshops.

### 4.3. Limitations

This study has several limitations. First, this was a cross-sectional and self-reported recognition survey of municipal government officers. Owing to the social desirability bias, we overestimated the status of awareness-raising activities in municipalities. Second, our findings might have underestimated the impact of the COVID-19 pandemic, as our participants had a larger population than all nationwide municipalities, and smaller municipalities may have been more affected by the COVID-19 pandemic. Regardless of these limitations, this is the first study to show the impact of the COVID-19 pandemic on municipal activities for ACP promotion. The findings from this study will help develop future policies for a mature ACP culture.

## 5. Conclusions

In response to the COVID-19 pandemic, Japanese municipalities canceled, scaled back, or changed their methods (other than face-to-face) to web-based, awareness-raising activities for EOL decision-making, particularly workshops for residents and HCPs. Although for workshops for HCPs, the face-to-face style was successfully substituted with a web-based style, more than half the workshops for residents were canceled due to the pandemic, and the transition to web-based workshops has not progressed. With the increased need for ACP during the COVID-19 pandemic, it is necessary to promote activities for residents, while sharing pioneering trials using information technology.

## Figures and Tables

**Table 1:** Characteristics of Municipalities (*n* = 433).

Variables		N or Median	% or IQR
Population (median, IQR)		48,091	20,494–116,624
Percentage of +65 aged population (median, IQR)		31.4	27.1–37.3
Financial capability index (median, IQR)		0.6	0.4–0.8
Municipality category (*n*, %)	City	279	64.4
	Ward	7	1.6
	Town	133	30.7
	Village	14	3.2
Number of hospitals per 10,000 population (median, IQR)		0.6	0.3–0.9
Number of clinics delivering home care per 1000 aged +65 population (median, IQR)		0.5	0.3–0.7
Number of home-visiting nursing agencies per 1000, aged +65 population (median, IQR)		0.3	0.2–0.4
Number of LTC facility beds per 1000, aged +65 population (median, IQR)		27.8	21.9–34.4
Percentage of home deaths (median, IQR)		14.2	11.4–17.2
Percentage of LTC facility deaths (median, IQR)		8.9	6.2–11.5
Focused anti-infection measures as of January 2022 (*n*, %)	Yes	372	85.9
	No	61	14.1

IQR: interquartile range.

**Table 2:** Percentage of municipalities that conducted awareness-raising activities and area comparison by the implementation of focused anti-infection measures.

Awareness-Raising Activity by Municipalities		Overall (*n* = 433)		Implementation of Focused Anti-Infection Measures ^¶^				
				Areas with Focused Anti-Infection Measures (*n* = 372)		Areas without Focused Anti-Infection Measures (*n* = 61)		*p*-Value for Comparisons between Areas with and without Measures *
		Number of Municipalities That Conducted Each Activity	% ^⁑^	Number of Municipalities That Conducted Each Activity	% ^†^	Number of Municipalities That Conducted Each Activity	% ^‡^	
(A) For community residents	Workshops for residents	369	85.2	323	86.8	46	75.4	0.020
	Individual consultation services	246	56.8	214	57.5	32	52.5	0.459
	Information to residents concerning healthcare and long-term care at the end-of-life	283	65.4	246	66.1	37	60.7	0.405
(B) For healthcare providers and care workers	Workshops for healthcare providers	248	57.3	219	58.9	29	47.5	0.097
	Workshops for care workers	276	63.7	244	65.6	32	52.5	0.048
	Discussion and review meetings among healthcare professionals, care workers, and local government officers	239	55.2	214	57.5	25	41.0	0.016

**^¶^** The focused anti-infection measures have been implemented from the beginning of January 2022 to the end of March 2022. * Comparisons between areas with and without focused anti-infection measures by Chi-squared tests. **^⁑^** The values represent the percentages among the 433 total municipalities. **^†^** The values represent the percentages in the 372 municipalities with focused anti-infection measures. **^‡^** The values represent the percentages in the 61 municipalities without focused anti-infection measures.

**Table 3:** Impact of the COVID-19 pandemic on activities among municipalities that conducted each activity and a comparison of the implementation of focused anti-infection measures **^¶^**.

Awareness-Raising Activity by Municipalities		Overall	Implementation of Focused Anti-Infection Measures ^¶^		
			Areas with Focused Anti-Infection Measures	Areas without Focused Anti-Infection Measures	*p*-Value for Comparisons between Areas with and without Measures *
		Number of Municipalities That Felt the Impact of the Pandemic/Number of Municipalities That Conducted Each Activity (%)	Number of Municipalities That Felt the Impact of the Pandemic/Number of Municipalities That Conducted Each Activity (%)	Number of Municipalities That Felt the Impact of the Pandemic/Number of Municipalities That Conducted Each Activity (%)	
(A) For community residents	Workshops for residents	317/369 (85.9%)	280/323 (86.7%)	37/46 (80.4%)	0.254
	Individual consultation support to residents for health and care at the end-of-life	42/246 (17.1%)	36/214 (16.8%)	6/32 (18.8%)	0.787
	Information to residents concerning healthcare and long-term care at the end-of-life	40/283 (14.1%)	35/246 (14.2%)	5/37 (13.5%)	0.907
(B) For healthcare providers and care workers	Workshops for healthcare providers	215/248 (86.7%)	192/219 (14.2%)	23/29 (79.3%)	0.213
	Workshops for care workers	232/276 (84.1%)	208/244 (85.3%)	24/32 (75.0%)	0.137
	Discussions and review meetings among healthcare professionals, care workers, and the local government	195/239 (81.6%)	173/214 (80.8%)	22/25 (88.0%)	0.382

**^¶^** The focused anti-infection measures have been implemented from the beginning of January 2022 to the end of March 2022. * Comparisons between areas with and without focused anti-infection measures by Chi-squared tests.

**Table 4:** Details of the qualitative analysis for the free description of the COVID-19 pandemic impact.

Details of Impact on the Activities			(A) For Community Residents		(B) For Healthcare Providers and Care Workers					
			Workshops for Residents (*n* = 369 Municipalities)		Workshops for Healthcare Providers (*n* = 248 Municipalities)		Workshops for Care Workers (*n* = 276 Municipalities)		Discussions and Review Meetings among Healthcare Professionals, Care Workers, and the Local Government (*n* = 239 Municipalities)	
Category	Sub-Category	Example of Codes	Number of Codes	% *	Number of Codes *	% *	Number of Codes *	% *	Number of Codes *	% *
Canceled	-	“canceled”	185	50.1	59	23.8	75	27.2	89	37.2
Conducted with some changes	Conducted face-to-face with some changes	“postponed”, “limited the number of participants”, “reduced the number of workshops”, and “excluded group discussions”	163	44.2	64	25.8	69	25.0	59	24.7
	Conducted by methods other than face-to-face	“web-based workshop”, and “distribution of DVDs”	96	26.0	154	62.1	163	59.1	121	50.6

* Multiple codes were extracted from one municipality. The percentages indicate the number of municipalities that conducted each activity.

## Data Availability

Data should not be shared owing to ethical issues.

## Appendix A

**Table A1:** Comparison between study participants and the national statistics.

Variables		All Municipalities in Japan (*n* = 1741)		Study Participants in This Study (*n* = 433)	
Population (median, 25 percentile–75 percentile)		23,690	7900–62,325	48,091	20,494–116,624
Percentage of population aged +65 (median, 25 percentile–75 percentile)		34.0	29.0–39.2	31.4	27.1–37.3
Financial capability index (median, 25 percentile–75 percentile)		0.5	0.3–0.7	0.6	0.4–0.8
Number of hospitals per 10,000 population (median, 25 percentile–75 percentile)		0.6	0–1.0	0.6	0.3–0.9
Number of clinics delivering home care per 1000, aged +65 population (median, 25 percentile–75 percentile)		0.4	0.2–0.7	0.5	0.3–0.7
Number of home-visiting nursing agencies per 1000, aged +65 population (median, 25 percentile–75 percentile)		0.2	0–0.4	0.3	0.2–0.4
Number of LTC facility beds per 1000, aged +65 population (median, 25 percentile–75 percentile)		30.5	22.9–40.6	27.8	21.9–34.4
Percentage of home deaths (median, 25 percentile–75 percentile)		12.7	9.3–16.3	14.2	11.4–17.2
Percentage of LTC facility deaths (median, 25 percentile–75 percentile)		8.4	5.2–12.1	8.9	6.2–11.5
Focused anti-infection measures, as of January 2022 (*n*, %)	Yes	1467	84.2	372	85.9
	No	275	15.8	61	14.1
